# Association between single nucleotide polymorphisms in the mu opioid receptor gene (*OPRM1*) and self-reported responses to alcohol in American Indians

**DOI:** 10.1186/1471-2350-9-35

**Published:** 2008-04-23

**Authors:** Cindy L Ehlers, Penelope A Lind, Kirk C Wilhelmsen

**Affiliations:** 1 From the Departments of Molecular and Experimental Medicine and Molecular and Integrative Neurosciences, The Scripps Research Institute, La Jolla, CA, USA; 2 Genetic Epidemiology Laboratory, Queensland Institute of Medical Research, Brisbane, Australia; 3 Departments of Genetics and Neurology, The Carolina Center for Genome Sciences and the Bowles Center for Alcohol Studies, University of North Carolina, Chapel Hill, NC, USA

## Abstract

**Background:**

Variation in response to the hedonic and adverse effects of a substance is in part an inherited factor that may influence its use, abuse and dependence. The mu opioid receptor is the primary site of action for opiates and individuals with polymorphisms in this receptor appear to have variation in the CNS effects of opiates. Several studies have suggested that this receptor may also mediate some of the effects of non-opioid drugs, such as alcohol. The purpose of this study was to investigate associations between 13 single nucleotide polymorphisms in the mu opioid receptor gene (*OPRM1*) with self-reported responses to alcohol, an endophenotype associated with the development of alcohol dependence, in American Indians living on eight contiguous reservations.

**Methods:**

Each participant gave a blood sample and completed a structured diagnostic interview. Additionally, response to alcohol was indexed using the expectation version of the subjective high assessment scale (SHAS-E). SNPs were genotyped in 251 participants and data analyses were conducted using SOLAR.

**Results:**

The estimated heritability (h^2^) for the SHAS-E phenotypes ranged from 0.01 to 0.28. Endorsing the expectation of a *more *intense response on one or more of the following items from the SHAS-E: buzzed, clumsy, dizzy, drunk, effects, high, nausea, sleepy, talkative, terrible, and/or uncomfortable after imbibing 2–3 drinks was significantly associated with having at least one minor allele for at least one of 7 SNPs (p < 0.01) in the *OPRM1 *receptor gene.

**Conclusion:**

These studies provide data to suggest that the minor allele, for most of the polymorphisms in the *OPRM1 *receptor gene investigated, was found to be associated with a more intense, and/or more adverse, response to alcohol, traits that are significantly correlated with lowered quantity of alcohol consumption and less susceptibility to dependence in this Indian population. These data further suggest that making conclusions on the role of the mu opiod receptor gene in the development of alcohol dependence may be limited if only one polymorphism in the gene is evaluated in isolation.

## Background

A number of studies have documented that the dosage requirements for targeted effects of CNS drugs can vary widely [1]. For example, in a study of over 3,000 patients experiencing pain following postoperative hip replacement, the therapeutic morphine dosage requirements varied almost 40-fold [2]. Wide inter-patient variability in response to, and therefore in the dosage requirement for morphine have been demonstrated in cancer patients receiving morphine for pain control [3].

It appears that a number of genetic and environmental factors can lead to significant variation in the doses of a drug necessary to produce therapeutic, hedonic and/or adverse effects. However, there is increasing evidence that gene polymorphisms may be an important factor in determining a person's sensitivity and tolerance to a drug. The mu opioid receptor (OPRM1) is the primary site of action for opiates; about 20 variants in the mu opioid receptor gene (*OPRM1*) have been identified with amino acid substitutions that have polymorphic frequencies over 1% [4-11]. The most common single nucleotide polymorphism (SNP) reported on is A118G (rs1799971), which encoded the Asp40Asn codon change with most data suggesting that it is a functional variant [4,12].

There have been a series of studies in both healthy volunteers and in clinical patients suggesting that, the A118G variant may alter response to opioid drugs (see [1] for review). Lotsch and colleagues reported the 118G allele conferred smaller analgesic effects and produced less pupillary constriction during morphine and morphine-6-glucuronide (MG6) infusion [13-15]. In an experiment using a measure of pain tolerance to electrical stimulation, higher MG6 concentrations were associated with a 25% increase in current (C25) participants with the 118G allele [16,17]. Similar findings have been found for alfentanil [18] and levomethdone [19]. In clinical studies, data from patients with the 118G polymorphism tend to confirm data from experimental pain studies where those patients with the variant required higher alfentanil doses for analgesia or more morphine during colorectal surgery [20] or for pain/toxicity associated with morphine use in renal failure [13,14]. However, it appears that the effects may be drug or disease specific owing to presumed variation in environmental and/or other uncontrolled variables [1,21,22].

Several studies have suggested that the mu receptor may also mediate some of the hedonic and/or addictive effects of non-opioid drugs, such as alcohol [23,24]. Indirect support for this hypothesis is provided by studies demonstrating the efficacy of naltrexone for the treatment of alcohol dependence [25-31]. Further support is provided by studies evaluating associations between response to naltrexone pharmacotherapy for alcohol dependence and the presence of the A118G variant. In a study that combined data from three different clinical trials, Oslin and colleagues [32] demonstrated that carriers of the 118G allele had a significantly lower rate of relapse and a longer time to a return to heavy drinking when compared to those individuals who were homozygous for the 118A allele. This finding was not supported in the Veterans Affairs (VA) Cooperative Study where no significant interactions were found between naltrexone treatment response and any polymorphic variants at each of the three opioid receptor genes [33]. More recently, data from the Study for the Combined Pharmacotherapies and Behavioral Interventions for Alcohol Dependence (COMBINE) study demonstrated that treatment with naltrexone produced a significantly improved clinical global outcome in alcohol dependent participants with the 118G allele, as compared to those with the 118A allele [34]. The A118G polymorphism has also been associated with an individual's response to a naloxone challenge with subjects with the 118G allele showing higher plasma cortisol concentrations [35,36].

There has been a plethora of studies that have investigated the relationship between a diagnosis of drug and/or alcohol dependence and the A118G polymorphism. The results have been conflicting and inconsistent. In a recent meta-analysis of 28 different studies, including over 8000 subjects, the conclusion was that the *OPRM1 *A118G variant did not appear to affect risk for substance dependence. However, the authors further speculated that additional research would be needed to determine whether another polymorphism in the gene might influence receptor function and thus risk for substance dependence [37]. An additional feature of these studies, that may have weakened the results, is the use of a dichotomous phenotype, drug dependence, a diagnosis that is made based on both heritable and non-heritable factors [38]. Town and colleagues [39] suggested that genetic studies on the influence of mu opioid receptors polymorphisms be viewed within the broader context of alcoholism where the opioid receptor genes are taken to be partial, rather than complete, risk factors for the disorder. Thus, it may be that polymorphisms in *OPRM1 *encode for a variant that influences a more narrowly defined risk factor for alcoholism. This risk factor is envisioned to partially influence the development of the disorder, but may or may not ultimately be associated with the diagnosis depending on the age of the participant, presence of other risk factors and environmental variables.

Individual sensitivity to alcohol represents such an inherited factor that affects the likelihood of drinking and mediates the disposition for developing alcoholism [40], and has a strong genetic basis [41]. In general, people at higher genetic risk for alcoholism are less sensitive to the effects of alcohol and people at lower genetic risk for alcoholism are more sensitive. Support for this theory is provided by many, but not all, studies examining the reaction to alcohol among children of alcoholics, who are at greatly elevated risk for developing alcoholism [42]. Results have indicated that at moderate doses of alcohol, subjects who are family history positive for alcoholism and subjects who are family history negative for alcoholism attain equivalent blood alcohol concentrations, but most studies have found that subjects with a positive family history rate themselves as significantly less intoxicated than control subjects with a negative family history [43-46]. Although not all studies agree [47], a meta-analysis focusing on subjective level of intoxication confirmed a diminished response to alcohol as a characteristic more frequently seen in subjects with a positive family history than in those with a negative family history [48]. In addition, an 8-year follow-up of previously studied men with positive and negative family histories found that both a family history of alcoholism and a low response to alcohol were related to the development of alcohol-related problems [49].

Studies using similar methodologies among groups at lower risk for alcoholism have provided additional support for the idea that individual sensitivity to alcohol might also mediate protection from developing alcoholism. Individuals of Asian heritage, who have mutations in the aldehyde dehydrogenase gene (ALDH2) [50-53], and individuals of Jewish decent [54], two groups with low rates of alcoholism, were found to have more intense, although not necessarily more negative, responses to alcohol than matched control subjects of average alcoholism risk.

Genetic studies of complex phenotypes, such as sensitivity to alcohol, often have advantages when they are conducted in well-defined populations such as Native American tribes living on reservations [55]. A once popular notion, called the firewater myth, proposed that Native American Indians are constitutionally predisposed to an altered response to drinking alcohol [56]. In one empirical study, Native American Indians, like Caucasian sons of alcoholics, were found to have less intense objective and subjective effects of alcohol in an alcohol challenge paradigm. Additionally, participants with at least 50% Native American heritage reported less intense effects of alcohol than did those with less than 50% Native American heritage, despite equivalent blood alcohol concentrations [57-59].

The present report is part of a larger study exploring risk factors for substance dependence among Native American Indians [57-68]. The lifetime prevalence of substance dependence in this Indian population is high and evidence for heritability and linkage to specific chromosome locations has been demonstrated [65,69-72]. The purpose of the present set of analyses was to determine if a significant association could be detected between 13 SNPs in the *OPRM1 *receptor gene and self-report of subjective response to alcohol in this population.

## Methods

Participants, who were of mixed heritage but at least one-sixteenth Native American, were recruited from eight geographically contiguous reservations with a total population of about 3,000 individuals. They were recruited using a combination of a venue-based method [73,74], and a respondent-driven procedure [75], as described previously [64,76]. To be included in the study a participant had to be an American Indian of one of four tribal groups between the age of 18 and 70 without major medical problems that would preclude mobility.

Potential participants gave written informed consent using a protocol approved for the study by The Institutional Review Board (IRB) of The Scripps Research Institute, the Scientific Advisory Committee of the GCRC, and the Indian Health Council, a tribal review group overseeing health issues for the reservations where recruitment was undertaken. They also responded to a screening questionnaire that was used to gather information on demographics, personal medical history, ethnicity and detailed measures of substance abuse history [77] and weight & height. Each participant also completed an interview with the Semi-Structured Assessment for the Genetics of Alcoholism (SSAGA) [78], which was used to make diagnoses [79]. Response to alcohol was assessed using the subjective high assessment scale-expectations version (SHAS-E). This scale consists of 14 items rated on Likert scales ranging from 0 (normal) to 36 (extreme effect). The participants indicated how intoxicated they felt after drinking 2–3 drinks for the following items: buzzed, clumsy, dizzy, drunk, effects of alcohol, energy, good, high, nausea, sleepy, talkative, uncomfortable, terrible overall and great overall. A total score was also calculated for the first 12 SHAS-E items. The intersession reliability of the SHAS, from which the SHAS-E was constructed, is approximately 0.80 with a Cronbach alpha of 0.96 overall [80]. The items cluster together with an overall item-to-total correlation of 0.80 or higher and a Cronbach alpha of 0.96. The Cronbach alpha for the SHAS-E is also 0.96 and values on the SHAS-E have been demonstrated to significantly (p < 0.0001) correlate with responses on the alcohol challenge SHAS at 30 and 60 minutes (r^2 ^= 0.49, 30 min, r^2 ^= 0.51, 60 min) (Schuckit and Smith, personal communication).

Two hundred (251) individuals have both genotype and phenotype data for this analyses. Power analyses revealed that for a medium effect size (0.5) that power at this n would be equal to 0.976. DNA was isolated from whole blood using an automated DNA extraction procedure. All primers, probes and reagents were purchased from ABI (Applied Biosystems, Foster City, CA). SNPs were genotyped using TaqMan™ fluorescence 5' exonuclease technology. Each 5 microL reaction contained 25 ng genomic DNA, 1.6× TaqMan assay primer/probe mix, 1× PCR Buffer A, 2.5 mM MgCl_2_, 250 microM dNTPs, and 0.5 U AmpliTaq Gold polymerase. Thermocycling was performed as recommended by ABI. Genotypes were determined on an ABI 7900 HT Fast Real-Time PCR System using the allelic discrimination mode. Hardy-Weinberg equilibrium analyses were completed in Haploview (version 4.0) [81].

**Figure 1:**
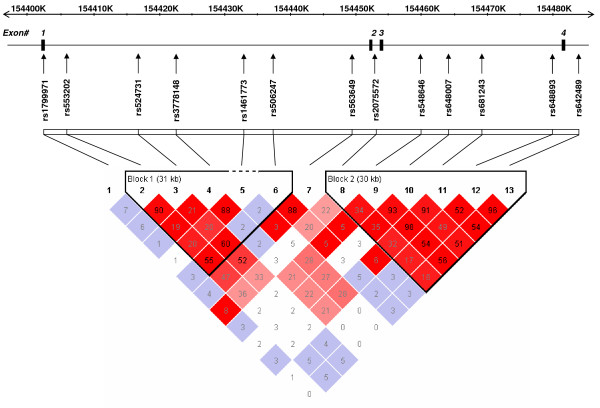
**A schematic representation of OPRM1 Gene Structure, Linkage**. Disequilibrium and Genotyped SNPs. The gene structure of OPRM1 is shown with exons numbered from 1 to 4 and relative exon size denoted by the width of the vertical bars. Thirteen SNPs analyzed in this study are shown in relation to their location across OPRM1. Linkage disequilibrium (LD; shown below the gene structure) data, as measured by the correlation coefficient r^2 ^statistic, was generated using Haploview [81]. LD causes tightly linked genetic variants to be highly correlated. Shading represents correlation magnitudes between low r^2 ^(white) and high r^2 ^(red).

Since genotype data was not available from the International HapMap Project public database[82] at the time this study was conceived (October 2004), seventeen single nucleotide polymorphisms (SNPs) in or near the *OPRM1 *locus were selected from the Applied Biosystems SNP database [83]. SNPs were initially chosen to be evenly distributed across *OPRM1 *with an average intermarker spacing of 5,133 bp. Assays for three SNPs (rs561720, hCV32237184, rs3798687) failed and were excluded from analyses. One SNP, rs12333298 in intron 1, showed significant (p = 0.018) deviations from Hardy-Weinberg equilibrium (HWE) at a p < 0.05 level and was also dropped from further analyses. The locations of the thirteen remaining SNPs typed in the study are shown in Figure 1 and SNP information, including the observed minor allele frequency (MAF), is described in Table 1.

**Table 1:** *OPRM1 *marker information, including genetic map position, location within *OPRM1 *and minor allele frequency.

Marker Name		Gene	Chromosomal	Functional	Alleles		MAF^d^					References^e^
				
dbSNP	Celera	Location	Location (bp)^a^	Location (bp)^b^	Minor^c^	Major	Indian	CEPH	China	Japan	Yoruba	
rs1799971	hCV8950074	Exon 1	154,402,490	+118	G	A	0.13	0.17	0.36	0.49	0.01	1,2,3,4,5,6,7,8,9,10
rs553202	hCV809975	Intron 1	154,406,510	+4,138	A	G	0.33	0.20	nd	nd	0.41	
rs524731	hCV809963	Intron 1	154,416,785	+14,413	A	C	0.32	0.18	0.04	0.02	0.05	1,3,4
rs3778148	hCV27499812	Intron 1	154,422,705	+20,333	T	G	0.08	0.14	0.04	0.02	0.04	
rs1461773	hCV8949980	Intron 1	154,433,062	+30,690	T	C	0.16	0.14	0.04	0.03	0.08	
rs506247	hCV27335981	Intron 1	154,437,620	+35,248	G	T	0.21	0.04	0.00	0.00	0.01	
rs563649	hCV809947	Intron 1	154,449,660	+47,288	A	G	0.24	0.08	0.13	0.06	0.16	1
rs2075572	hCV1691815	Intron 2	154,453,697	+51,325	G	C	0.39	0.43	0.20	0.21	0.62	1,2,3,4
rs548646	hCV3073603	Intron 3	154,459,840	+57,468	T	C	0.18	0.33	0.07	0.13	0.46	1,3
rs648007	hCV1691794	Intron 3	154,464,304	+61,932	T	C	0.18	0.33	0.07	0.13	0.46	1
rs681243	hCV3073596	Intron 3	154,469,434	+67,062	A	G	0.18	0.24	0.06	0.14	0.43	
rs648893	hCV3073587	Intron 3	154,480,321	+77,949	C	T	0.11	0.20	0.05	0.10	0.04	1,3,4,5
rs642489	hCV3073582	3' UTR	154,484,368	+81,996	A	C	0.11	0.21	0.04	0.10	0.13	

^a^bp = base-pair position on NCBI Genome Build 127.

^b^Position relative to transcription start site at 154,402,372 on Chromosome 6 (NCBI Genome Build 127).

^c^The allele with the lowest frequency.

^d^MAF = Minor Allele Frequency for the Indian tribes studied (Indians) and the reference populations CEPH (European), China, Japan, Yoruba (African). *nd *= no data.

^e^Reports where each SNP has previously been genotyped: 1. Xuei et al., 2007 [100]; 2. D. Zhang et al., 2007 [98]; 3. H. Zhang et al., 2006 [99]; 4. L. Zhang et al., 2006 [101]; 5. Gelernter et al., 2007 [33]; 6. Luo et al., 2003 [97]; 7. Smith et al., 2005 [102]; 8. Crowley et al., 2003 [103]; 9. Shi et al., 2002 [104]; 10 Y. Zhang et al. 2005 [12].

The OPRM1 region spanned by the 13 SNPs analyzed in this study is 81.8 kb. This region includes 140 SNPs that were typed by the HapMap project. Because the allele frequencies observed in this Indian population for each of the SNPs is intermediate between the allele frequencies observed for the European (CEU) and Asian (JC) HapMap populations, we investigated the linkage disequilibrium structure in these populations. The inferred CEU and JP population haplotypes were used estimate the consensus phylogenic tree based on 500 bootstrapped trees produced with Neighbor-Joining method based on Kimura distance matrix as implemented in PHYLIP [84].

The inferred haplotypes for the 140 available SNPs for CEU and JP populations can be divided into four major clades, which can be further subdivided into related clades some of which may arisen from recombination of the primary clades. Each of the subclades characteristically is relatively specific to either the CEU or JP population based on additional SNPs that were typed in by the HapMap project. It was found using haplotype analysis that the SNPs typed in the present study represented the major haplotypes identified in HapMap but they were unable to identify the large number of minor haplotypes. Ultimately, a more complete analysis depends on resequencing the OPRM1 gene and determining which sequence variants have functional significance.

The total additive genetic variance (heritability, h^2^) and its standard error were estimated for the SHAS-E phenotypes using SOLAR [85]. A genetic association analyses was conducted where the number of copies of the minor allele of each individual was used as a covariate in a variance component analysis as implemented in SOLAR v2.0.4 [86], and the statistical significance of the ability of the covariate to explain phenotypic variance was determined. Age and sex were also accounted for in the analyses. To account for multiple comparisons, in these exploratory analyses, nominal significance was set at the p < 0.01 level.

## Results

The demographic characteristics of the sample are virtually equivalent to the U.S. census data for these tribes and have been presented previously [65,87]. The mean age of the sample was 30.2 (± 0.7) yrs, there were 110 males and 141 females with a mean of 11.5(0.1) yrs of education, 60% of the sample was over 50% Native American heritage as estimated by their federal Indian blood quantum and 55% reported income of less than $20,000 per annum. Within the sample of 251 participants 160 (64%) were found to have a lifetime DSM-III-R diagnosis of alcohol dependence and an additional 47(18.7%) were found to have alcohol abuse. One hundred seventy-three (69%) were current drinkers who reported a mean of 13 years of alcohol use. The mean number of drinking occasions reported per month was 10 and their mean drinks per occasion were nine.

In total, 13 *OPRM1 *SNPs spanning a region of 81.9 Kb were genotyped in 251 individuals. DNA from these individuals had been previously genotyped for linkage analyses [64,70,71]. The 251 individuals originated from a total of 41 families, comprising of 1 to 4 generations, with an average number of seven members per family (range, 1–30). The 251 individuals within the 41 families that were genetically informative includes: 77 parent-child, 212 sibling, 26 half sibling, 8 grandparent-grandchild, 151 avuncular, and 245 cousin relative pairs [65]. Marker information including genetic map position, location within *OPRM1*, and minor allele frequencies within this Indian population (as well as four reference populations for comparison) are listed in Table 1. Mendelian inconsistencies were identified using PEDSTATS [88] and made up 0.03% of the data. The physical locations of and pattern of linkage disequilibrium (LD) between the 13 SNPs typed across the *OPRM1 *gene are schematically presented in Figure 1.

The estimated heritability (h^2^) for the SHAS-E phenotypes ranged from near to zero for "energy" to .28 for "terrible" (see Table 2). The only two phenotypes with significant heritability were talkative and terrible (p < 0.01). Table 2 also gives values for the mean ± S.D. for each of the SHAS-E items as well as the total for this population. As seen in Table 3, endorsing a more intense response on one or more of the following SHAS-E items: dizzy, drunk, high, nausea, talkative, and/or uncomfortable after imbibing 2–3 drinks was significantly associated with having at least one minor allele for 7 SNPs (p < 0.01) in or near the OPRM1 receptor gene. Whereas, for the 118G allele, the most commonly genotyped Asn40Asp polymorphism, there was only a trend for an association with reporting a less intense response to alcohol for the items: dizzy (p < 0.02) and sleepy (p < 0.02).

**Table 2:** Estimated heritability (h^2^) for the Subjective High Assessment Scale-Expectations (SHAS-E) phenotypes.

Item	Trait	Mean	Std Dev	Range	Heritability	Std. Err.	p
1	Buzzed	12.32	11.48	0–36	0.09	0.10	0.18
2	Clumsy	9.52	10.19	0–36	0.01	0.09	0.45
3	Dizzy	7.75	10.44	0–36	0.09	0.10	0.18
4	Drunk	8.27	10.73	0–36	0.14	0.10	0.06
5	Effects of Alcohol	12.15	11.67	0–36	0.15	0.11	0.07
6	Energy	9.24	9.45	0–35	0		0.50
7	Good	12.33	10.40	0–36	0.05	0.10	0.29
8	Great	11.54	10.57	0–36	0.06	0.11	0.29
9	High	11.65	10.44	0–36	0.01	0.09	0.44
10	Nausea	6.0	9.85	0–36	0.12	0.11	0.11
11	Sleepy	7.20	9.99	0–36	0.23	0.12	0.02
12	Talkative	13.31	11.22	0–36	0.26	0.12	**0.0089**
13	Terrible	7.95	11.03	0–36	0.29	0.11	**0.002**
14	Uncomfortable	9.67	10.06	0–36	0.05	0.11	0.31

	Total	118.80	98.7	0–377	0.11	0.11	0.14

Mean, standard deviation and range of values are given. Significant values (p < 0.01) are highlighted in bold.

**Table 3:** Association of *OPRM1 *SNPs with response to alcohol, as measured by the Subjective High Assessment Scale-Expectations (SHAS-E) questionnaire. Significant values (p < 0.01) are highlighted in bold.

Marker	Minor	Increaser	Subjective High Assessment Scale-Expectations Item														
	
	Allele	Allele	Buzzed	Clumsy	Dizzy	Drunk	Effects	Energy	Good	Great	High	Nausea	Sleepy	Talk	Terrible	Uncom	Total
rs1799971	G	A	0.26	0.84	0.02	0.23	0.67	0.64	0.98	0.49	0.20	0.32	0.02	0.67	0.71	0.07	0.24
rs553202	A	A	0.03	0.10	**0.01**	**0.001**	0.10	0.48	0.32	0.64	**0.001**	**0.01**	0.15	0.21	**0.008**	**0.008**	0.02
rs524731	A	A	0.03	0.10	**0.01**	**0.001**	0.09	0.56	0.26	0.94	**0.002**	0.06	0.13	0.16	**0.01**	0.07	0.02
rs3778148	T	T	0.02	0.02	**0.01**	**0.01**	0.02	0.92	0.39	0.52	**0.000**	**0.003**	0.09	**0.001**	0.11	**0.000**	**0.003**
rs1461773	T	T	0.02	0.03	**0.01**	**0.03**	0.03	0.94	0.67	0.60	**0.003**	**0.01**	0.07	**0.005**	0.08	**0.009**	**0.01**
rs506247	G	G	0.40	0.95	0.51	0.06	0.91	0.58	0.63	0.55	0.35	0.44	0.47	0.48	0.07	0.83	0.64
rs563649	A	A	0.73	0.77	0.56	0.12	0.70	0.91	0.73	0.39	0.84	0.62	0.58	0.38	0.06	0.57	0.91
rs2075572	G	G	0.12	0.04	**0.01**	**0.003**	0.31	0.69	0.76		0.21	**0.002**	0.05	0.74	0.07	0.17	0.06
rs548646	T	T	**0.01**	**0.01**	**0.001**	0.02	0.02	0.12	0.77	0.91	0.06	0.02	0.04	0.03	0.33	0.02	**0.005**
rs648007	T	T	0.09	0.02	**0.01**	0.08	0.10	0.21	0.85	0.95	0.12	**0.01**	0.23	0.09	0.82	0.05	0.03
rs681243	A	A	**0.02**	**0.01**	**0.001**	0.02	0.03	0.15	0.90	0.91	0.05	**0.006**	0.06	0.06	0.37	0.02	**0.007**
rs648893	C	C	0.51	0.34	0.37	0.62	0.45	0.09	0.85	0.82	0.78	0.82	0.60	0.28	0.42	0.50	0.41
rs642489	A	A	0.43	0.25	0.31	0.55	0.40	0.09	0.83	0.80	0.79	0.71	0.45	0.28	0.50	0.55	0.36

The increaser allele for the TOTAL phenotype (i.e., the allele associated with a higher TOTAL score)

TOTAL = sum of scores reported for the first 12 SHAS-E items.

## Discussion

The CNS effects of alcohol range from mild euphoria (high), to impaired coordination, to ataxia, decreased mentation, labile mood, to poor judgment, slurred speech, nausea and vomiting, and finally to respiratory failure, coma and death, depending on the dose imbibed [89]. The final level of impairment appears to depend on a number of factors including a persons' gender, age, weight, prior experience with alcohol and level of tolerance [40]. Another source of variation in response to alcohol is individual variation in alcohol metabolism. Some individuals, particularly East Asians who are homozygous for the ALDH2*2 allele, are intolerant of alcohol and report intense facial flushing, tachycardia, hypotension, headache, nausea and vomiting following drinking more than one drink [67]. African Americans, with at least one ADH1B*3, also report expecting to have a more intense response to a standard dose of alcohol when compared to African Americans who are homozygous for the ADH1B*1 allele [90].

Other sources of the genetic variation in sensitivity and tolerance to alcohol not attributed to differences in alcohol metabolism are less well understood. Several studies have found moderate heritability for level of response to alcohol. In one study, heritability was found to be 60% for a composite sensitivity measure that was used during an alcohol challenge in twins [91]. Correlations of level of response to alcohol using body sway and the SHAS in an alcohol challenge paradigm using sibling pairs as participants was reported to be 0.36 [41]. Lower correlations were found in first-degree relatives using a retrospective self-report measure to assess level of response to alcohol (0.12–0.22). In the present study, values for the heritability of the SHAS-E were found to range from near to zero (for energy) to 0.28 (for terrible).

Further evidence for a genetic component to level of response to alcohol was provided by a genome-wide segregation analysis that evaluated subjective response to alcohol challenge in sibpairs. In that study, nine chromosome regions with LOD scores between 2.2 and 3.2 suggesting potential regions of interest in the genome that may contribute to the variance in alcohol responsivity [41]. An expanded dataset, collected in the same laboratory, also identified five areas of the genome with LOD scores between 2.2 and 2.6 for level of response to alcohol in sibpairs [92]. None of the locations identified in those studies were on chromosome 6 (6q24q25) near the location of the mu opioid receptor. However, a previous study in this Indian population found suggestion for linkage on chromosome 6q24q25 for several substance dependence phenotypes, as well as Body Mass Index, suggesting genes in that location may be associated with risk for substance dependence and other consumption-related phenotypes [71].

In the present study, evidence was obtained for an association between expectations of the effects of a standard dose of alcohol and polymorphisms in the *OPRM1 *receptor gene. Participants with at least one 118G allele for the Asp40Asn polymorphism reported that they expected to feel a less intense response to alcohol for the items: dizzy (p < 0.02) and sleepy (p < 0.02) when compared to individuals without any 118G alleles, findings that were not significant in these analyses when multiple comparisons were taken into account. These data are, however, consistent with data from Kim and colleagues [93], who found that alcoholics with two copies of the 118G allele spent more days drinking than those who were heterozygous or homozygous for the 118A allele, perhaps suggesting a less intense response to alcohol. Assuming that alcohol may act as a partial agonist at the mu opioid receptor, the findings in the present study of a trend for reduced effect of alcohol in participants with the 118G allele, are also consistent with studies that evaluated response to opioid agonists where a reduced response to drug challenge (pupillary diameter, pain, respiratory depression) and/or increased dosage requirements are seen in those individuals with the 118G allele (see [1] for review).

Few studies have evaluated whether an association exists between response to alcohol and polymorphisms in the *OPRM1 *gene. In one study, the ability of naltrexone to blunt an alcohol-induced high was found to be greater in those participants with the 118G allele [94]. The finding of a more intense response to a mu opioid receptor antagonist found by Ray and Hutchinson [94] is consistent with previous studies that have demonstrated that subjects with the 118G allele that were given naloxone had higher cortisol concentrations [36]. It is also consistent with the finding that naltrexone may be more efficacious for the treatment of alcoholism in those with at least one 118G allele [32]. However, Ray and Hutchinson [94,95] have also reported that young participants in an IV alcohol challenge, with one 118G allele, reported feeling *more *subjective feelings of "high" across rising breath alcohol concentrations, as compared to those participants homozygous for the 118A allele. These findings are not consistent with the findings in the present study for the 118G allele, nor are they particularly consistent with studies that have found a less intense response to opioid agonists. However, the findings of Ray and Hutchinson [94,95] are consistent with the findings in the present study of an association between expecting to experience a more intense response to alcohol and carrying at least one minor allele for eight other SNPs in the opioid receptor gene. Since, in the study of Ray and Hutchinson [94,95], only one SNP was genotyped and the ethnic characteristics of the sample were not specified, it is possible that the findings reflected stratification of the sample or that the A118G variant was in linkage disequilibrium with several other alleles that may encode for a more intense response to alcohol. These data further suggest that making conclusions on the role of the mu opioid receptor gene in the development of alcohol-related behaviors may be limited if only one polymorphism in the gene is evaluated in isolation.

Several alcohol- or drug-related association studies [96-99] have expanded their investigations to include up to 20 SNPs in or near *OPRM1*, although all include the A118G variant. Ide and colleagues [96] genotyped 20 SNPs including 10 SNPs in the 3'UTR region among Japanese subjects meeting ICD-10 criteria for methamphetamine (MAP) dependence/psychosis and controls. Four SNPs (including the A118G and rs2075572 variants that were genotyped in the present study) representing the major haplotypes observed in the study sample were tested for association with four features of MAP dependence/psychosis. While A118G and two other SNPs were not associated with MAP dependence/psychosis, the rs2075572 G-allele was significantly associated with increased risk for a diagnosis of MAP dependence/psychosis (p = 0.011), as well as four aspects/symptoms of the disorder (p < 0.01). Interestingly, within this Indian population, the rs2075572 G-allele was related to expecting to feel a more intense response to alcohol in four of the 14 items of the SHAS-E, an indication that carriers of this allele may be protected from developing alcohol dependence. Zhang and colleagues [98] investigated the relationship between heroin-induced subjective responses in a Chinese population and ten SNPs selected throughout *OPRM1*. They found three SNPs in intron 1 were associated with an increased risk of positive responses on first use of heroin and were likely contributing to further heroin consumption. However, A118G and rs2075572 were not associated with any differences in heroin-induced subjective responses. In another study, Luo and colleagues [97] typed eight variants in alcohol, cocaine and opioid and poly-substance dependent European Americans (EA) and African Americans (AA). They found that the A-allele of the -2044C/A polymorphism was a susceptibility allele for the combination of alcohol and opioid dependence in the EA sample, but not the AA sample. Once again, A118G was not associated with any of the substance dependent phenotypes. Finally, Zhang and colleagues [99] studied the role of *OPRM1 *genetic variation in a large case-control sample of alcohol dependent and/or drug (cocaine and/or opioid) dependent European Americans. Thirteen SNPs, five of which were typed in the present study, were genotyped representing the major haplotypes observed in HapMap. Seven SNPs (but not A118) were associated with alcohol, cocaine, opioid plus opioid/cocaine dependency. Zhang and colleagues [99] found that the frequencies of the rs524731 A-allele and rs648893 T-allele were significantly higher among the dependent EA subjects. Within this Indian population, the rs524731 A-allele and rs648893 T-allele were generally associated with a more intense response to alcohol.

## Conclusion

In conclusion, these data represent the first association analysis of a level of response to alcohol phenotype with multiple SNPs in the *OPMR1 *receptor gene in American Indians. SNPs highlighted in prior studies of substance dependence phenotypes were also identified as well as new SNPs of potential importance to substance dependence research. The results of this study should, however, be interpreted in the context of several limitations. A more conservative approach to multiple comparisons would have led to fewer significant effects. Level of response to alcohol was evaluated using the SHAS-E, and a more direct measure of intoxication using the SHAS or body sway may produce more reliable results. Haplotype analysis using the SNPs typed in this study was unable to specially tag all of the clades observed in the HapMap population. Ultimately, a more complete analysis depends on resequencing the OPRM1 and determining which sequence variants have functional significance. Additionally, the findings may not generalize to other Native Americans or represent all Indians of the tribes studied, and comparisons of association findings to non-Indian populations may be limited by differences in a host of potential genetic and environmental variables. Finally, because this population has significant admixture estimates of allele frequencies may produce biased results. Despite these limitations, this report represents an important step in an ongoing investigation to understand the genetic determinants associated with the development of substance use disorders in this high risk and understudied ethnic group.

## List of abbreviations

CNS: Central Nervous System; DNA: Deoxyribonucleic Acid; ADH1B*3: Alcohol Dehydrogenase 1B* 3; ADH1B*1: Alcohol Dehydrogenase 1B*1.

## Competing interests

The authors declare that they have no competing interests.

## Authors' contributions

CLE contributed to the recruitment, collection and analysis of the clinical and genetic data on the subjects. KCW contributed to the genetic and heritability analyses. PAL did the genotyping and its analysis. All authors contributed to writing the paper.

## Pre-publication history

The pre-publication history for this paper can be accessed here:


